# Effects of HIF-1*α* and HIF2*α* on Growth and Metabolism of Clear-Cell Renal Cell Carcinoma 786-0 Xenografts

**DOI:** 10.1155/2010/757908

**Published:** 2010-06-27

**Authors:** Swethajit Biswas, Helen Troy, Russell Leek, Yuen-Li Chung, Ji-liang Li, Raju R. Raval, Helen Turley, Kevin Gatter, Francesco Pezzella, John R. Griffiths, Marion Stubbs, Adrian L. Harris

**Affiliations:** ^1^Weatherall Institute of Molecular Medicine, University of Oxford, John Radcliffe Hospital, Oxford OX3 9DU, UK; ^2^CR UK Tumour Pathology Group, Nuffield Department of Clinical Laboratory Sciences, University of Oxford, John Radcliffe Hospital, Oxford OX3 9DU, UK; ^3^Northern Institute for Cancer Research (NICR), Newcastle University, Freeman Hospital, Newcastle-Upon-Tyne NE7 7DN, UK; ^4^CR UK Biomedical Magnetic Resonance Research Group, Division of Basic Medical Sciences, St. George's, University of London, London SW17 0RE, UK; ^5^CRUK Clinical Magnetic Resonance Research Group, Institute of Cancer Research, Sutton, Surrey SM2 5NG, UK; ^6^CRUK Cambridge Research Centre, Li Ka Shing Centre, Robinson Way, Cambridge CB2 ORE, UK

## Abstract

In cultured clear-cell renal carcinoma (CCRCC) 786-0 cells transfected with HIF1*α* (HIF-1+), HIF-2*α* (HIF-2+), or empty vector (EV), no significant differences were observed in the growth rates *in vitro*, but when grown *in vivo* as xenografts HIF-2*α* significantly increased, and HIF-1*α* significantly decreased growth rates, compared to EV tumors. Factors associated with proliferation were increased and factors associated with cell death were decreased in HIF-2+ tumors. Metabolite profiles showed higher glucose and lower lactate and alanine levels in the HIF-2+ tumors whilst immunostaining demonstrated higher pyruvate dehydrogenase and lower pyruvate dehydrogenase kinase 1, compared to control tumors. Taken together, these results suggest that overexpression of HIF-2*α* in CCRCC 786-0 tumors regulated growth both by maintaining a low level of glycolysis and by allowing more mitochondrial metabolism and tolerance to ROS induced DNA damage. The growth profiles observed may be mediated by adaptive changes to a more oxidative phenotype.

## 1. Introduction

The HIF*α* transcription factors, HIF-1*α* and HIF-2*α*, mediate adaptive responses to tumor hypoxia, as well as regulating an extensive transcriptional response involving the induction of genes for angiogenesis, glucose metabolism/cellular energetics, cell growth, metastasis, apoptosis, and extracellular matrix (ECM) remodelling [1]. HIF-1*α* and HIF-2*α*, despite some overlapping effects, can uniquely regulate distinct genes [2]. HIF-1*α* for example is primarily involved in glucose metabolism by upregulating glycolytic enzymes [3, 4] whilst limiting pyruvate uptake by the mitochondria [5, 6] and down regulating the electron transport chain (ETC) activity by altering the subunit composition of COX, minimising ROS generation [7]. In contrast, HIF-2*α* is uniquely involved in tumor growth and cell cycle progression through interaction with c-Myc [8, 9]. 

The most direct link between genetic events that predispose to cancer and activation of the HIF pathway is observed in tumors associated with inactivation of the von Hippel-Lindau (VHL) tumor suppressor gene, particularly VHL-associated clear-cell renal cell carcinoma (CCRCC) (for review see [10]). The pseudohypoxic VHL-defective CCRCC cells show a bias toward HIF-2*α*, and overproduction of HIF2*α* (but not HIF1*α*), has been found to be sufficient to override the tumor suppressor function of VHL in xenograft studies [11–13]. HIF-2*α* expression (in a mouse model of CCRCC) is necessary for the development of the typical clear-cell phenotype, demonstrating the important role of HIF-2*α* in CCRCC [14].

Using retroviral transfection in 786-0 cells, Raval et al. [12] confirmed that exogenous expression of HIF-1*α* upregulated transcriptional products involved in pH regulation (CAIX) and cell fate (BNIP3), whereas expression of HIF-2*α* upregulated a different set of proteins which were involved in cell proliferation (cyclin D1), cell growth (TGF-*α*), and angiogenesis (VEGF). However, the *in vitro* studies demonstrated no differences in the proliferation rate between 786-0 cells that either exogenously expressed HIF1*α* (HIF-1+), overexpressed HIF-2*α* (HIF-2+) or were infected with empty vector (EV) (control cells). But when these manipulated 786-0 cells were grown *in vivo* as xenografts, a different tumor growth profile emerged showing that HIF-2*α* caused significantly *increased *growth rates and HIF-1*α* caused significantly *decreased* growth rates when compared to EV tumors. Similar findings (HIF-2*α* facilitating tumor growth *in vivo*) have also been made in two nonepithelial tumors, teratoma [13] and neuroblastoma [15] *in vivo*. 

However, apart from the growth curves, few studies have been reported that investigate the role of HIF-2*α* in *epithelial* cancers *in vivo* (for review see [16]). Most epithelial cancer cells rely on HIF-1*α* transcriptional products to mediate tumor metabolism including the effect first described by Warburg [17] more than 80 years ago that leads to the reprogramming of tumor cells from mitochondrial respiration to aerobic glycolysis (see [18–20] for review). The human VHL -/- clear-cell renal cancer cell line, 786-0, provides a model for investigating the effects of both HIF*α* isoforms, particularly HIF-2*α*, on tumor growth and metabolism *in vivo*, since it constitutively expresses *only* HIF-2*α*. To further understand the role of HIF-2*α* *in vivo*, we have investigated the effects of HIF-2*α* expression not only on CCRCC 786-0 tumor growth, but also on metabolic adaptation to tumor progression by using Magnetic Resonance Spectroscopy (MRS) methods both noninvasively *in vivo* and *ex vivo* on tumor extracts.

The rationale for using 786-0 line in our experiments is that CC-RCC comes in two HIF types that is HIF-2 only and HIF-2 + HIF-1. Therefore, using a CC-RCC, HIF-2 only expressing cell line is appropriate to investigate the role of HIF-1 in CC-RCC tumour growth/biology as well as the effects of HIF-2 overexpression on an *endogenous* HIF-1 null background (see [21]). Expression of HIF-2*α* resulted in a significant increase in tumor growth rate similar to that observed previously [12] whilst HIF1+ tumors grew even more slowly than EV tumors. Immunohistochemistry (IHC) was chosen rather than Western Blotting because the necrosis and heterogeneity of *in vivo* tumors causes poor reproducibility. IHC allows heterogeneity to be scored and the extent of protein expression to be determined in a semiquantitative fashion. Using a combination of immunostaining and/or ^1^H or ^31^P Magnetic Resonance Spectroscopy (MRS), we also demonstrate that expression of HIF-2*α* decreased the expression of HK-II, LDH5 and pyruvate dehydrogenase kinase 1 (PDK1) with a concomitant increase in pyruvate dehydrogenase (PDH) expression. This, together with higher glucose and lower lactate and alanine levels found in the HIF-2+ tumors (compared to both EV and HIF-1+ tumors), results in a more oxidative, DNA damage-tolerant phenotype that supports enhanced tumor growth, similar to the Sporadic VHL-deficient clinical subtypes of CCRCC described by Gordan et al. [21].

## 2. Materials and Methods

### 2.1. Human Clear-Cell Renal Cancer Cell Lines

Stable polyclonal pools of G418 selected 786-0 renal cancer cells expressing the relevant HIF*α* isoform (HIF-1+) were produced *in vitro* from the same pool as previously described [12]. Polyclonal pools retrovirus-infected with the pBMN-Z-IRES-Neo-based HIF-1*α*, HIF-2*α* (HIF-2+), or control (EV), were released by trypsinization and subsequently resuspended in PBS.

### 2.2. Xenografts

786-0 cells (1 × 10^7^) transfected with HIF-1or 2*α* or control in 100 *μ*l PBS were injected subcutaneously into the dorsal flanks of *nu/nu *mice. Three cohorts were generated. Two consisting of 21 mice with 7 in each group (HIF-1+, HIF 2+ and EV) for growth, immunohistochemistry and histology; and one consisting of 15 mice with 5 in each group (HIF-1+, HIF 2+ and EV) which were grown to at least 500 mm^3^, the minimum size possible for *in vivo* Magnetic Resonance Spectroscopy experiments. 

Tumor size was measured bidirectionally in all experiments, twice per week using calipers, with the longest dimension (l) and shortest dimension (s) measured postimplantation. Tumor volume (mm^3^) was calculated by measuring length, width and depth using callipers and 1 ∗ w ∗ d ∗ (*π*/6). 

#### 2.2.1. Tumor Processing

 The mice from cohort (1 and 2) were sacrificed by cervical dislocation at day 42, the tumors bisected with one-half snap-frozen for storage at −80°C, and the other half-embedded in paraffin. The mice from cohort (3) were anesthetized with a single i.p. injection of a Hypnovel/Hypnorm/water (1 : 1 : 2) mixture as previously described [22] prior to the MR experiment (details below). At the end of the experiment the tumors were freeze-clamped and stored at −80°C. Subsequently the frozen tumors were extracted in 6% perchloric acid, as previously described [23] and the neutralized extracts were freeze-dried and reconstituted in 1 ml deuterium oxide for high resolution MRS. Cyrostat sectioning of frozen tumors was performed for mouse CD31 staining as previously described [24], and Fuhrman's criteria were applied to histological grading for characterization of nucleoli morphology [25].

#### 2.2.2. Immunohistochemistry

 Tumors were prepared for immunohistochemistry as follows. Briefly, endogenous tissue peroxidase activity was blocked using two drops of 0.3% hydrogen peroxide (1 : 100 dilution of 30% H_2_O_2_ stock (BDH Laboratory Supplies, Poole, UK) in distilled water), to cover each section. After two rounds of immersion in PBS, for 5 minutes each, 2.5% normal horse serum (Normal horse serum concentrate—Vector Laboratories Inc, California, USA, diluted in PBS) was applied to each section for 30 minutes at room temperature to prevent nonspecific primary antibody binding.

*Primary antibodies* were directed against HIF-1*α*, HIF-2*α* and CAIX; mouse monoclonals (ESEE122), (237/B5) and (M75), respectively, University of Oxford. Ki67, Cyclin D1: mouse monoclonals (MIB-1) and (DSC-6), and GLUT-1: rabbit polyclonal; DAKO, Ely, UK: Cleaved caspase-3; rabbit monoclonal, R+D Systems, UK; BNIP3: mouse monoclonal (ANa40) SIGMA, UK; CD10: mouse monoclonal (56C6), Abcam, UK; VEGF: SP28, rabbit monoclonal antibody, Abcam,UK; Phospho-Ser_473_ Akt and PTEN: rabbit monoclonals 736E11 and 138G6 respectively, Cell Signalling, USA; Hexokinase-II: rabbit polyclonal, Chemicon, USA; LDH5: sheep polyclonal, Abcam, UK; PDH E2 Complex: mouse monoclonal (15D3), Invitrogen, USA; PDK-1: goat polyclonal, Santa Cruz, USA; TOM-20: mouse monoclonal (F10), Santa Cruz, USA; PGC-1 *β*: rabbit polyclonal, Santa Cruz, USA; *γ*H2AX (phospho-Ser_139 _ Histone H2A.X): Rabbit Monoclonal, 20E3, Cell Signaling, USA; 8-Hydroxyguanosine (8-OH-G): goat polyclonal, Alexis Biochemicals, Nottingham, UK; OGG1 Rabbit polyclonal (ab204) Abcam, UK.

The Envision-HRP ChemMate polymer kit (DAKO, Ely, UK) was used for detection of either mouse and rabbit monoclonal or rabbit polyclonal primary antibodies, as per the manufacturer's instructions. Where applicable, the relevant secondary antibodies of antigoat (P0160, DAKO, Ely, UK), antisheep (ab6747, Abcam, UK) and antirat (P0450, DAKO, Ely, UK) were used.

The majority of primary antibodies were detected with a 3,3-diaminobenzidine (DAB+) chromogenic substrate system as part of the Envision kit. Nuclei were counterstained with Haemotoxylin before mounting onto plastic coverslips with AquaMount (Gurr GmBH, Strasbourg, Germany). For Ki67 (MIB-1 clone) detection, ChromogenSG (Vector Laboratories Inc., California, USA) was used and the nuclei counterstained with Nuclear Fast Red (Sigma-Aldrich, St. Louis, USA). Sections treated in both these ways were dehydrated through methanol and xylene, before haemotoxylin counterstaining and mounting with DPX.

#### 2.2.3. Assessment of Tumor Immunostaining

 Each tumor section was assessed blindly and independently by two observers. Photomicrographs were taken at x100 hpf. Semi-quantitative analysis of protein expression was performed using a modified “Histoscore” method, as previously described [26]. For Ki67 and Cleaved caspase-3 scoring, positive and negatively stained cells within 5 individual tumor areas, consisting of 100 cells each, were scored [27]. Tumor necrosis was quantified as the % area of tumor replaced by necrosis, as identified by light microscopy [28]. CD31^+^ Chalkley Vessel Count (CVC) was the average value from the three fields [29].

### 2.3. Magnetic Resonance Spectroscopy (MRS)

 Anesthetized mice were placed in the bore of a Varian 4.7 T nuclear magnetic resonance (NMR) spectrometer at St. George's University of London, and tumors were positioned in the center of a 15-mm two-turn ^1^H surface or ^ 31^P MRS coil. Voxels were selected from scout gradient echo images, and localized shimming yielded linewidths of the order of 20–30 Hz. The PRESS localization method with water suppression with a repetition time of 2 seconds was used to detect choline [30]. For ^31^P MRS, image selected *in vivo* spectroscopy (ISIS) [31] localised spectra of tumors were acquired. MRUI software was used for all spectral processing programs, including preprocessing, fitting and quantification of peak areas of the observed metabolites. 

^1^H MR spectra of the neutralised tumor extracts were obtained using a Bruker 600 MHz spectrometer (pulse angle 45°; repetition time, 3.5 seconds). The water resonance was suppressed by gated irradiation centred on the water frequency. 25 *μ*l of 10 mM Sodium 3-trimethylsilyl-2,2,3,3-tetradeuterpropionate (TSP) was added to the samples for chemical shift calibration and quantification. The pH was re-adjusted to pH 7 prior to ^1^H MRS.

#### 2.3.1. Statistics

 For analysis of the immunohistochemical expression of individual proteins between all tumor groups, the nonparametric Kruskal-Wallis (ANOVA) test was used. Results from one cohort were displayed as histograms with standard error of the mean (SEM) in the figures. Dunn's post hoc test for all data sets was calculated if *P* < .05 and displayed in the figures. Immunohistochemical protein expression between specific pairs of 786-0 tumor groups was compared using the Mann-Whitney unpaired *t-*test, where mentioned in the text. The Spearman rank testing was used to demonstrate correlations between non-parametric variables. For the MRS data a two-tailed *t* test was used for significance levels. Significant results were designated if *P* < .05.

## 3. Results

### 3.1. Effect of Transfection of Specific Retroviral HIF*α* Isoform in CCRCC 786-0 Xenografts

**Figure 1:**
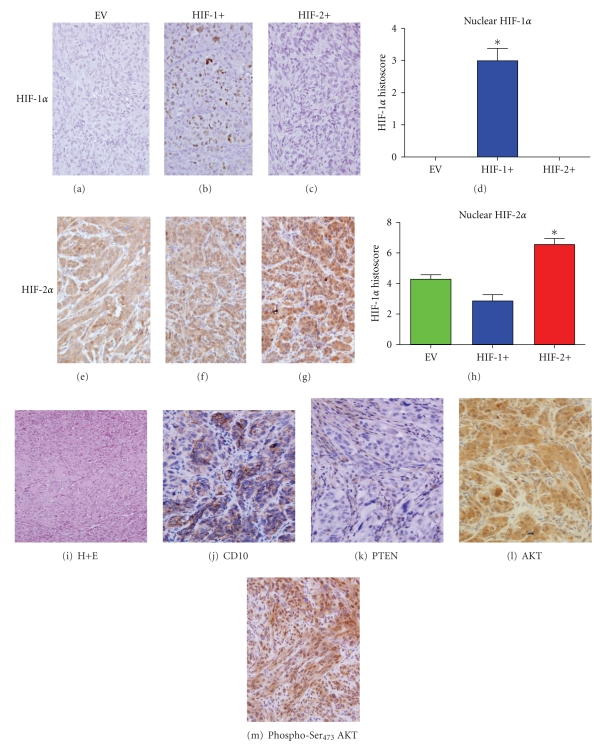
Features of CCRCC 786-0 tumors. (a–d) (x100 hpf) HIF-1*α* nuclear staining only (*P < .0001). (e–h) Differential HIF-2*α* nuclear staining (*P = .0004). (i) Clear-cell/sarcomatoid morphology (x40 hpf). (j) CD10^+^ staining typical of clear-cell RCC lineage (x100 hpf). (k) PTEN staining; positive staining only identified in murine fibroblasts (x100 hpf). (l) Akt staining, (m) Phospho-Ser_473_ Akt staining (highest in HIF-2+ tumors). *P values were calculated using an ANOVA test.

We have previously demonstrated [12] that the appropriate HIF*α* isoform protein is expressed after retrovirally-mediated infection of specific HIF*α* isoform constructs within a bicistronic IRES-neo cassette, *in vitro*. These *in vitro* findings were confirmed *in vivo* in tumors grown subcutaneously as xenografts in mice in all three groups. HIF-1*α* expression was identified only in the HIF-1+ tumors, and only within the nuclear compartment, whereas the EV and HIF-2+ tumors showed no staining for HIF-1*α* (Figures 1(a)–1(d)). 

However, HIF-2*α* expression was identified in both nuclear and cytoplasmic compartments in all 3 tumor groups (Figures 1(e)–1(h)), but showed a significant increase only within the nuclear compartment of HIF-2+ tumors (Figure 1(g)). No significant changes in HIF-2*α* expression were seen in either compartment of the HIF1+ or the EV tumors (Figures 1(e)–1(f)). A further cohort (not shown in figures) of *in vivo* tumors confirmed the findings of HIF-1 and HIF-2*α* expression as well as a similar growth pattern in the 3 tumor types.

### 3.2. Effect of HIF*α* Isoform Expression on Grade and Phenotype of CCRCC 786-0 Xenografts

21 tumors were evaluated after H+E staining, and each exhibited a high Fuhrman's tumor grade of either 3 or 4, with the majority of tumors demonstrating sarcomatoid dedifferentiation. There were no differences in Fuhrman's grade between the three tumor groups (a representative example is shown in Figure 1(i)). Only one tumor (an EV tumor) was morphologically wholly clear-cell and one tumor (HIF-2+) was completely replaced with sarcomatoid de-differentiation which is a clinically recognised variant of high grade tumors. However despite their sarcomatoid de-differentiation the tumours retained expression of typical clear-cell renal cancer markers such as CD10 (Figure 1(j)), pancytokeratin and vimentin positive expression, on a CK-7 negative background (data not shown). 786-0 cells were PTEN negative (Figure 1(k)), but the surrounding murine fibroblasts demonstrated positive staining. This finding was confirmed by the high levels of phospho-Ser_473_ (activated) Akt expression in the 786-0 cells of the EV tumors (Figure 1(l) and 1(m)). Expression of activated Akt was increased in the HIF-2+ tumors compared to EV and HIF-1+ tumors. This may be because expression of TGF*α* was increased in the HIF-2+ tumors with a similar increase in activated EGFR (Tyr_1173_-EGFR) expression, compared with EV and HIF-1+ tumors (data not shown).

### 3.3. Effect of HIFa Expression on Tumor Proliferation and Apoptosis in 786-0 Xenografts

**Figure 2:**
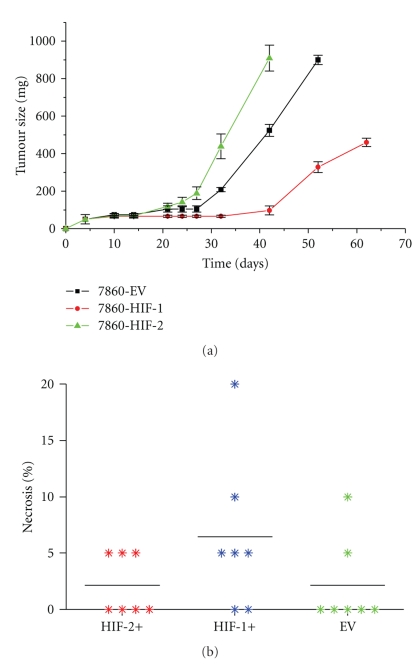
(a) Growth curve kinetics of 786-0 CCRCC tumors in vivo. (b) Areas of tumor necrosis (%) *P > .1. *P values were calculated using an ANOVA test.

In contrast to the growth patterns *in vitro* where the 3 cell types demonstrated similar proliferation rates [12], growth patterns *in vivo* showed that there were significant differences between HIF-1+, HIF-2+ and EV 786-0 tumors (Figure 2(a)). The differences in overall growth between the 3 tumor groups were dependent on the lag phase for each tumor group as well as the rate of tumor growth. The HIF-2+ tumors had the shortest lag phase (21 days) followed by the EV tumors (27 days) with the HIF-1+ tumors taking the longest time (>32 days). Once the lag phase was over, the actual rates of growth were 45 ± 5.4 mm^3^/day for HIF-2+ (*P* = .09 compared to EV), 35 ± 3.3 mm^3^/day for EV tumors (*P* = .0007 compared to HIF-1) and 18 ± 4.0 mm^3^/day for HIF-1+ tumors. There were no significant differences in necrosis between the different tumor types (Figure 2(b)).

**Figure 3:**
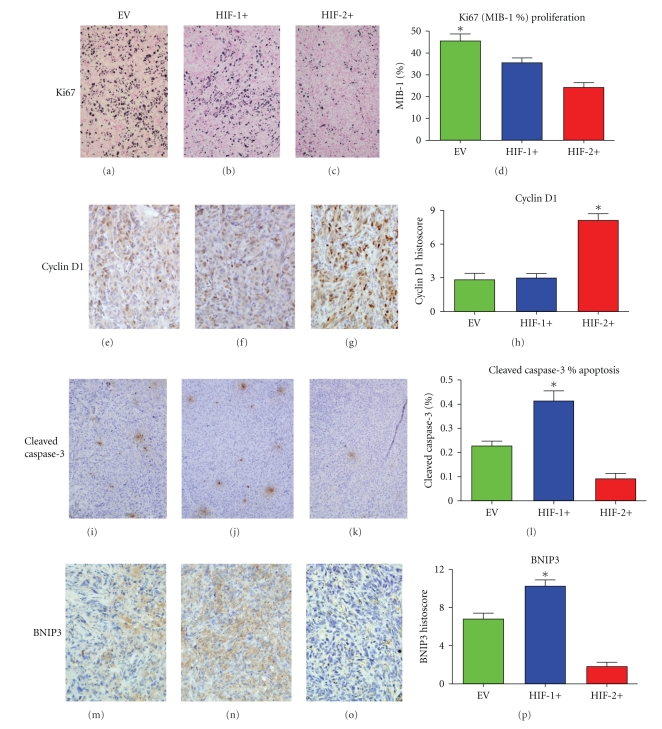
(x100 hpf). Growth and death markers in CCRCC 786-0 tumors. (a–d) Ki67 proliferation index (*P = .0006). (e–h) Cyclin D1 expression (*P = .001). (i–l) Apoptosis as measured by cleaved-caspase-3% index (*P = .0002). (m–p) BNIP3 expression (cytoplasm only) (*P = .0002). *P values were calculated using ANOVA test.

The EV tumors demonstrated the highest Ki67 (MIB-1%) proliferation rates in comparison to both the HIF-1+ and HIF-2+ tumors (*P* = .0006) (Figures 3(a)–3(d)). Cyclin D1 expression however was highest in the HIF-2+ tumors (*P* = .0010) (Figures 3(e)–3(h)). The overall rates of apoptosis measured by cleaved-caspase 3 were very low (<0.5%) in all the tumor groups (Figure 3(i)–3(l)). The HIF-1+ tumors had the highest rate of apoptosis (~0.4%) compared to controls (*P* = .0002), whereas the HIF-2+ tumors had only 0.1% compared to the EV tumors with ~0.25%. Because the apoptotic rates were so low, we also considered potential regulators of alternative death pathways, such as BNIP3, which has been implicated in cancer cell autophagy [32, 33]. The intergroup expression of BNIP3 demonstrated that HIF-2+ tumors showed the lowest expression and HIF-1+ tumors the highest (*P* = .0002) (Figures 3(m)–3(p)). However, as previously mentioned there were no significant differences in the level of tumor necrosis between the different groups (Figure 2(b)).

### 3.4. Effect on Factors Related to Glucose Metabolism; Glut-1, HKII, LDH

**Figure 4:**
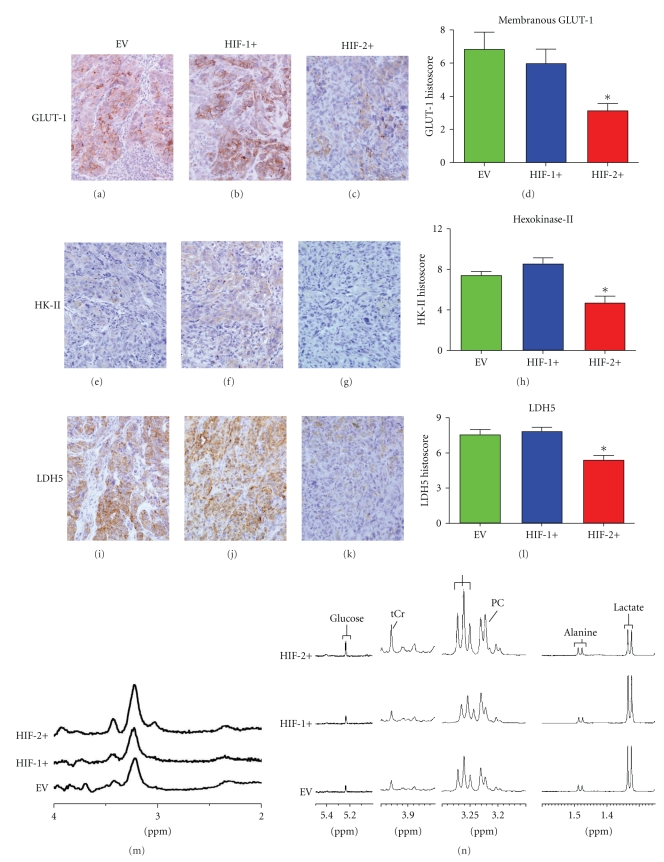
Metabolism-related markers and metabolic profiles of CCRCC 786-0 tumors (x100 hpf). (a–d) GLUT-1 expression(*P = .01). (e–h) Hexokinase-II expression (*P = .0006). (i–l) LDH5 expression (*P = .004), (m) *In vivo* ^1H^MRS of 786-0 tumors. (n) High-resolution ^1H^MR Spectra of tumor extracts. *P values were calculated using an ANOVA test.

Expression of GLUT-1 was attenuated in the HIF-2+ tumors (Figures 4(a)–4(d)) in comparison to the other two tumor groups (*P* = .01), in contrast to the *in vitro* findings by Raval et al. [12]. Expression of two glycolytic enzyme proteins, HK-II (Figures 4(e)–4(h)) and LDH5 (Figures 4(i)–4(l)), was significantly lower in the HIF-2+ tumors in comparison to both the HIF-1+ and EV tumors, whereas there was no difference in the expression of these glycolytic enzymes between HIF-1+ tumors and EV tumors.

### 3.5. Metabolites Measured by ^1^H MRS and ^31^P of 786-0 Xenografts and in Tumor Extracts

^1^H MRS of *in vivo* tumors demonstrated higher levels of free choline (which resonates at ~3.2 ppm) in the HIF-2+ tumors (Figure 4(m)) compared to HIF-1+ and EV tumors. After *in vivo* scanning the tumors were freeze-clamped and metabolites were measured at high field in tumor extracts (which gives better resolution than *in vivo*) by ^1^HMRS (Figure 4(n) and Table 1). The MR spectra shown in Figure 4(n) are representative samples of various spectral regions of the high resolution spectra obtained from the extracts of each tumor type. The significantly higher levels of choline/phosphocholine (PC) found in extracts of HIF-2+ tumors reflected the raised choline found in the tumors *in vivo* by ^1^H MRS. *In vivo* ^31^PMRS of the tumors showed no significant differences between the parameters ATP, PME, PDE, Pi (data not shown). Using Pi spectral shift analysis [34], similar values for intracellular pH (pHi) were found in all 3 tumor groups. Similar to the *in vivo* results, no differences were observed in the high energy phosphates (ATP+ADP) between the different tumor types (Table 1).

Signals from glucose, creatine (tCr), and taurine were also significantly higher in the HIF-2+ tumors, whereas alanine and lactate were significantly lower compared to the HIF-1+ and EV tumors. This is more clearly demonstrated in the detailed analysis of the metabolites shown in Table 1 and described below. These data imply a more oxidative and less glycolytic phenotype for the HIF-2+ tumors.

### 3.6. Effects on Factors Related to Mitochondrial Regulation and Free Radical Damage; PDH, PDK-1, TOM-20, 8-OH-Guanosine and OGG1

**Figure 5:**
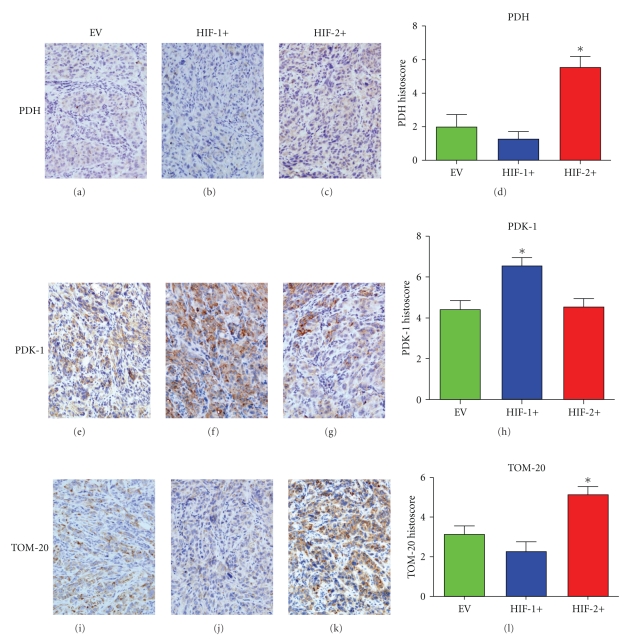
Markers of oxidative phosphorylation and mitochondrial load in CCRCC 786-0 tumors. (a–d) PDH expression (*P = .003). (e–h) PDK-1 expression (*P = .006). (i–l) TOM-20 (mitochondrial marker) expression (*P = .004). *P values were calculated using an ANOVA test.

PDH (Figures 5(a)–5(d)) was upregulated and PDK-1 (Figures 5(e)–5(h)) down-regulated in the faster growing HIF-2+ tumors. Higher expression levels of the cellular mitochondrial load marker, TOM-20 (Figures 5(i)–5(l)) was also seen in the HIF-2+ tumors and in turn this was mirrored by an increase in expression of the mitochondrial biogenesis regulator, PGC-1*β* (data not shown). Overall, this is consistent with an increase in mitochondrial biosynthesis and activity. 

**Figure 6:**
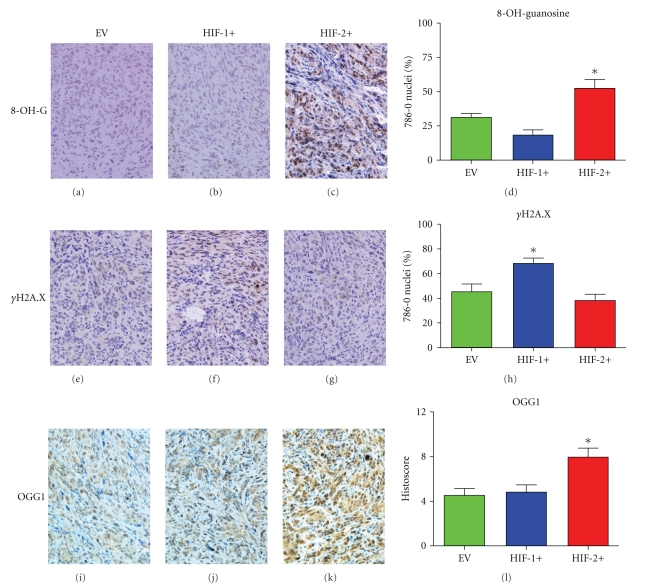
Oxidative stress and DNA damage/repair in CCRCC 786-0 tumors. (a–d) 8-OH-Guanosine staining (oxidative stress marker) (*P = .001). (e–h) *γ*H2A.X staining (double-stranded DNA damage) (*P = .004). (i–l) OGG1 expression (*P = .006). *P values were calculated using an ANOVA test.

The HIF-2+ tumors were also under a comparatively greater degree of oxidative stress, as manifest by higher levels of 8-OH-guanosine staining compared to the other two tumor groups (Figures 6(a)–6(d)). However immunostaining of *γ*H2A.X (Figures 6(e)–6(h)) showed lower levels indicating less DNA damage in HIF2+ than in HIF1+ or EV tumors. Expression of OGG1 (a DNA repair enzyme) was higher in the HIF-2+ tumors compared to HIF-1+ and EV tumors (Figures 6(i)–6(l)).

### 3.7. Effects on Factors Related to Neoangiogenesis

**Figure 7:**
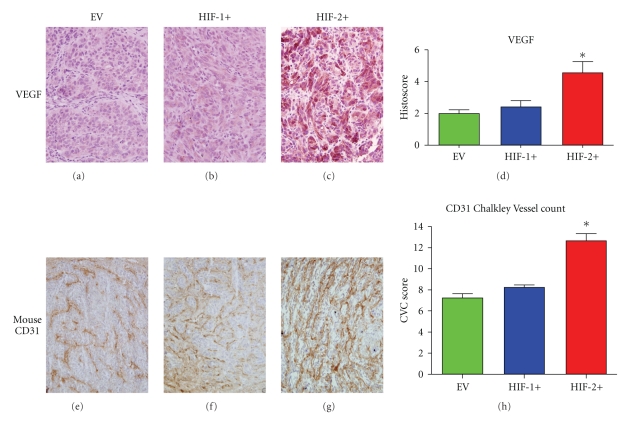
Markers of angiogenesis in CCRCC 786-0 tumors. (a–d) VEGF expression (*P = .003). (e–h) Murine CD31^+^ vessel staining (*P = .001). *P values were calculated using an ANOVA test.

VEGF, identified only in the cytoplasm of tumor cells, was higher in the HIF2+ tumors compared to HIF1+ and EV tumors (Figures 7(a)–7(d)). The Chalkley Vessel Count (CVC) using an anti-mouse CD31 antibody, was also higher in the HIF-2+ tumors compared to both HIF-1+ and EV groups (Figures 7(e)–7(h)), which was consistent with the pattern of VEGF expression.

## 4. Discussion

The tumor grade of 786-0 tumors does not alter with differential HIF*α* isoform expression on a HIF-2*α*-only expressing background, whether grown as cultured cells or as xenografts that demonstrate a high grade phenotype and characteristic morphology. Although the patterns of HIF*α* isoform expression *in vivo* were similar to those found in the CCRCC 786-0 cells *in vitro* [12], there were some differences between the levels of specific transcription factors expressed *in vitro* and *in vivo.* The expression of BNIP3, cyclin D1, TGF*α* and VEGF in the *in vivo* model were similar to HIF*α* isoform expression found *in vitro. *However the expression of GLUT-1 was comparatively lower in the HIF-2+ tumors *in vivo* (see below for discussion) consistent with a more oxidative phenotype. 

### 4.1. Tumor Growth and Related Death Pathways

This *in vivo* study showed that the growth of CCRCC 786-0 tumors was biphasic, with an initial growth lag phase followed by growth acceleration. The HIF-1+ tumors, which were overall the slowest growing of the three groups, had the longest lag phase whereas the EV tumors started to grow at day 27, and the HIF-2+ tumors at day 21. The lag times and growth rates *in vivo* were similar to those observed previously [12]. These differences in early growth may reflect stress of a poor blood supply which could have affected early establishment of the tumors, since the HIF-2+ tumors had the highest levels of CD31^+^ angiogenesis and VEGF, but the shortest initial growth lag phase compared to EV and HIF-1+ tumors. 

Tumor growth is a balance between cellular proliferation and cell death. The increased levels of cyclin D1, an important regulator of cell cycle progression, were seen in the faster growing HIF-2+ tumors, but surprisingly they had the lowest proliferation index (Ki67) and very low levels (<0.5%) of apoptosis *in vivo.* This may be the result of two independent background factors. Activated Akt is constitutively expressed in the 786-0 xenografts, due to the PTEN -/- status, facilitating tumor growth [35] and an antiapoptotic phenotype [36]. Since the levels of necrosis were similar between tumor groups, alternative cell death mechanisms, such as autophagy, were considered to explain the differences in growth between the tumor types. BNIP3 levels were significantly lower in HIF2+ tumors *in vivo,* and were consistent with the *in vitro* results of Raval et al. [12] showing that over-expression of HIF-2*α* attenuated BNIP3 expression. Since both HIF-1+ and EV tumors had significantly higher levels of BNIP3, and since their levels of apoptosis were very low, we hypothesize that BNIP3 induces autophagic cell death in this 786-0 model as a default death mechanism. In addition, phosphocholine and glycerophosphocholine were highest in the HIF2+ tumors compared to HIF-1+ and EV tumors. Usually (although not always [37]) high levels of PC and GPC are associated with increased proliferation and growth, but in the present study the HIF-2+ tumors had lower proliferation (Ki67) but higher growth rates, compared to controls. The findings in the HIF-2+ tumors combined with low apoptosis and autophagy are in contrast to the tumor suppressor effects reported in both neuroblastoma [38] and colon cancer [39] xenograft models, as well as a rat GS9L orthotopic glioma model [40].

This discrepancy in the growth profile between the 786-0 CCRCC model and other non-CCRCC model systems may lie in the different HIF*α* backgrounds of the parental cell lines which are different. The 786-0 CCRCC cell line only expresses HIF-2*α*, whereas both the N1E-115 neuroblastoma cell line [38] and the SW480 colon cancer line [39] endogenously expressed HIF-1*α*, as well as HIF-2*α*. It is the expression of HIF-1*α* in both of these other model systems that is thought to facilitate tumor growth, and over-expression of HIF-2*α* antagonises this effect. Similarly, in the rat GS9L orthotopic model, the tumor suppressive effect of HIF-2*α* over-expression was caused by apoptosis [40]. 

However in the CCRCC 786-0 model, we suggest that over-expression of HIF-2*α* regulates growth both by maintaining some glycolysis, albeit at a lower level, allowing more mitochondrial metabolism (higher PDH, lower PDK) and tolerance to DNA damage (*γ*H2A.X) resulting from increased ROS (8-OH-guanosine) production. 

A recent study by Gordan et al. [21] raises the possibility that HIF1*α* acts as a tumor suppressor, and our data showing decreased growth rate of the HIF1+ compared to EV tumors seem to support this suggestion [10].

### 4.2. Tumor Metabolism and Its Consequences

In non-CCRCC cells *in vitro*, Akt signalling has also been demonstrated to positively regulate glycolysis in a HIF-1*α* independent manner [41] mainly through mediating the localization of GLUT-1. HIF-2+ tumors had lower expression of GLUT-1 in comparison to the EV tumors, despite supranormal levels of activated Akt. These findings are in contrast to the *in vitro* findings of Raval et al. [12] who demonstrated that HIF-2*α* was the principal regulator of GLUT-1 expression. An explanation for this discrepancy between the *in vitro *and *in vivo* results could be that GLUT-1 expression is also sensitive to changes in intracellular glucose concentration. Higher concentrations of glucose were found in the HIF-2+ tumors, and could have attenuated GLUT-1 localization. This higher tumor glucose level along with decreased expression of HK-II and LDH5 and lower levels of lactate and alanine in the HIF-2+ tumors compared to both the EV and HIF-1+ tumors, suggested a decreased glycolytic flux in HIF2+ tumors compared to HIF-1+ and EV tumors. However HIF-1*α* (in an endogenous HIF-2*α*-only background) *in vivo*, appeared to have no effect on GLUT-1 expression since there were no differences between glucose concentrations and GLUT-1 expression in HIF-1+ and EV tumors. Interestingly, Cyclin D1 (which was higher in the HIF2+ tumors) has been shown in an *in vivo* mouse mammary cancer model to reduce the expression of both HK-II and LDH5 [42].

HIF-1 modulates multiple key metabolic pathways to optimize use of O_2_ and glucose in response to changes in availability of these substrates, in order to most efficiently generate ATP without excessive generation of ROS [7]. PDH is the key enzyme that determines whether pyruvate formed during glycolysis from glucose will be metabolised to lactate or oxidised in the TCA cycle. Its regulator, PDK, has been shown to be expressed in a HIF-1*α* dependent manner [5, 6]. PDK negatively regulates PDH by phosphorylation, and in EV tumors the level of aerobic glycolysis was characterised by high PDK and low PDH indicating the basal level of glycolysis in these tumors. A similarly high PDK, low PDH was also found in HIF-1+ tumors, suggesting that the basal level of aerobic glycolysis in EV 786-0 cells *in vivo* cannot be increased by exogenous expression of HIF-1*α*; alternatively this may be due to mutually interacting effects of the pVHL -/- [43] and PTEN -/- status [44] of the parental 786-0 cell line. 

In HIF-2+ tumors, in contrast, PDK-1 was decreased and PDH was increased suggesting that the HIF2+ tumors rely on a less glycolytic, more oxidative metabolism. We hypothesize that increased oxidation would supply more reducing equivalents for the electron-transport chain (ETC), increase mitochondrial O_2_ consumption and thus increase the ATP supply to support the greater growth rate of the HIF2+ tumors. In support of this hypothesis were the findings of higher levels of TOM-20 (mitochondrial load) and lower BNIP3 levels in HIF-2+ tumors, consistent with a higher mitochondrial mass, less mitophagy, and up-regulation of respiration, the converse of what was found with HIF-1*α* expression [45]. 

Since activated Akt is known to have the paradoxical effect of increasing mitochondrial O_2_ consumption and subsequently facilitating ROS generation [46], it could be postulated that the supranormal levels of activated Akt status and the metabolic shift to greater oxidative metabolism in the HIF-2+ tumors is mainly responsible for the higher levels of 8-OH-Guanosine immunostaining (high ROS stress) identified in these tumors. In spite of high ROS, *γ*H2A.X levels and OGG1 indicated resistance to DNA damage in the HIF2+ tumors. These findings (summarised in Table 2) are in agreement with Gordan et al. [8, 9] who have shown that HIF-2*α* promotes cell cycle progression by enhancing c-Myc mediated cyclin D2, leading to enhanced growth and resistance to DNA damage. This was not achieved by modulating c-Myc levels, but by its interactions with partners. Although we did not stain for c-Myc in the 786-0 xenografts, it is highly likely that HIF-2*α*-mediated enhancement of c-Myc activity played a role in the xenografts studied here.

## 5. Conclusions

Tumor metabolism represents the end point of many signal cascades recruited by oncogenic activation. HIF*α* isoforms, particularly HIF-1*α*, have been shown to be key regulators of aerobic glycolysis in cancer cells. This is because HIF-1*α* not only mediates the transcription of cytoplasmic glycolytic enzymes and PDK-1, which phosphorylates and inactivates PDH, but also attenuates mitochondrial function by down regulating ETC activity, leading to a consequent reduction of oxidative phosphorylation [7]. However, it could be speculated that expression of HIF-2*α*, in manipulated CCRCC 786-0 tumors, overcomes the HIF-1*α* effects, which results in a more oxidative tumor phenotype that supports a more aggressive phenotype. These results (see Table 2) are in general agreement with the recent findings of Gordan et al. [8] who showed that HIF-2*α* expression in pVHL- deficient CCRCC tumor lines potentiated c-Myc activity, resulting in enhanced growth and resistance to replication stress. We propose that the growth profiles observed in the HIF-1+ and HIF-2+ tumors that we have studied may be mediated by HIF-1*α* inhibition of the c-Myc oncoprotein (slowing HIF-1+ growth) whilst HIF-2*α* potentiates c-Myc transcriptional activity (HIF-2+) and promotes tumor growth by an adaptive change to a more oxidative phenotype. In addition, the overall results from our study are consistent with the findings of Gordan et al. [21] who showed that clinical CCRCC tumors expressing only HIF-2*α* were bigger in size and more resistant to replicative stress compared to those that expressed *both* HIF-1*α* and HIF-2 *α*. This may define a critical role for HIF-2*α* in the biology of VHL -/- CCRCC enabling greater growth; this demonstrates that in certain contexts HIF-1*α* can act as a tumor suppressor (see also [10, 47]).

## Figures and Tables

**Table 1:** Metabolite levels measured by ^1^H MRS in 786-0 tumor extracts.

Metabolite	EV	HIF-1+	HIF-2+
Leucine	0.13 ± 0.01	0.17 ± 0.02	0.17 ± 0.01^a^
Iso Leucine	0.06 ± 0.01	0.08 ± 0.005	0.08 ± 0.005^a^
Lactate	5.13 ± 0.85	5.43 ± 0.51	2.54 ± 0.58^a,b^
Alanine	0.84 ± 0.06	0.88 ± 0.05	0.63 ± 0.04^a,b^
Choline	0.17 ± 0.02	0.22 ± 0.04	0.29 ± 0.05^a^
PC	0.73 ± 0.09	0.66 ± 0.14	1.14 ± 0.05^a,b^
Taurine	13.96 ± 1.55	13.02 ± 0.92	16.70 ± 0.66^b^
Cr	1.26 ± 0.14	1.46 ± 0.15	2.00 ± 0.17^a,b^
Glucose	0.63 ± 0.10	0.73 ± 0.12	1.19 ± 0.29^a^
ATP+ADP	0.91 ± 0.20	0.95 ± 0.13	1.14 ± 0.15

Metabolites expressed as *μ*mol/g wet weight tissue (*n* = 3–5). ^a^denotes statistically significant different from EV and ^b^denotes statistically significant difference (*P* < .05) from HIF-1+. A two-tailed *t* test was used for significance levels.

**Table 2:** Overview of molecular characteristics of HIF-1*α* and HIF-2*α* expression on CCRCC 786-0 xenografts compared to EV xenografts.

Parameter	Marker	HIF-1+	HIF-2+
Growth	volume	⇊	⇈
	Ki67	↓	↓
	Cyclin D1	—	⇈
Apoptosis	Caspase-3	<0.5%	<0.5%
Autophagy	BNIP3	↑	⇊
Glycolysis	GLUT-1	—	⇊
	HK II	—	⇊
	LDH5	—	⇊
	Lactate	—	⇊
	Glucose	—	⇈
Mitochondrial Respiration	PDH/PDK	↓	⇈
	TOM-20	—	⇈
ROS	8-OH-guanosine	↓	↑
DNA	*γ*H2A.X	↑	⇊
damage/repair	OGG1	—	⇈
Angiogenesis	VEGF	—	⇈
	CD31	—	⇈

## Abbreviations

CCRCC: Clear-cell renal cell carcinoma
EV: CCRCC 786-0 tumors grown from cells retrovirally infected with empty vector.
HIF-1+: CCRCC 786-0 tumors grown from cells retrovirally infected with expression of HIF-1*α*.
HIF-2+: CCRCC 786-0 tumors grown from cells retrovirally infected with HIF-2*α*.
